# Serial passage through resistant and susceptible cucumber cultivars affects the virulence of *Fusarium oxysporum* f. sp. *cucumerinum*

**DOI:** 10.1002/mbo3.641

**Published:** 2018-05-23

**Authors:** Xiaoqing Huang, Manhong Sun, Xiaohong Lu, Shidong Li

**Affiliations:** ^1^ Institute of Plant Protection Chinese Academy of Agricultural Sciences Beijing China

**Keywords:** *Fusarium oxysporum* f. sp. *cucumerinum*, resistant cultivar, serial passage, susceptible cultivar, virulence variation, virulence‐related gene

## Abstract

*Fusarium oxysporum* f. sp. *cucumerinum* (Foc) is the causal pathogen of cucumber Fusarium wilt resulting in losses to cucumber production. To investigate the effects of the selective pressures of host plants on the virulence of Foc, a low virulence isolate, foc‐3b, was successively inoculated on resistant and susceptible cucumber cultivars for five generations. The virulence of the original isolate diverged; virulence was significantly strengthened after serial passage on the resistant cultivar and weakened on the susceptible plants (*p* ˂ .05). The expression of four virulence‐related genes of *F. oxysporum*, G‐protein α subunit gene *fga1*, sucrose nonfermenting 1 gene *snf1*, F‐box protein gene *frp1*, and Class V chitin synthase gene *chsV*, was quantified using real‐time PCR. All genes were significantly upregulated after serial passage on the resistant cultivar, compared to the original strain, and the expression of *snf1* was downregulated in strains re‐isolated from the susceptible plants (*p* ˂ .05). A significant positive correlation was found between the expression levels of gene *snf1*,* frp1*, and *chsV* and disease severity of cucumber Fusarium wilt, suggesting these genes may impact virulence differentiation. This study will improve the management of cucumber Fusarium wilt and provide insight into the mechanisms underlying virulence of *F. oxysporum*.

## 1 INTRODUCTION

*Fusarium oxysporum* is a ubiquitous soil‐borne pathogenic fungus that consist of more than 150 formae speciales that specialize on a range of plant species (Fourie, Steenkamp, Ploetz, Gordon, & Viljoen, 2011; Lievens, Hanssen, & Rep, 2012). *Fusarium oxysporum* f. sp. *cucumerinum* Owen (Foc) is a forma specialis capable of infecting the vascular bundle of cucumber, leading to necrotic lesions on the stem base, foliar wilting, and eventually plant death (Owen, 1955, 1956). Foc has been isolated in planting areas around the world and confirmed as a serious threat to cucumber production (Hu et al., 2010; Jenkins & Wehner, 1983; Martínez, Aguilar, Guirado, Álvarez, & Gómez, 2003).

Three races of Foc, race 1, 2, and 3, have been proposed based on the reaction of a cultivar to the disease (Armstrong, Armstrong, & Netzer, 1978). A total of 11 vegetative compatibility groups (VCGs) have been identified according to the ability to form heterokaryons, among which at least five are found in China (Vakalounakis & Fragkiadakis, 1999; Vakalounakis, Wang, Fragkiadakis, Skaracis, & Li, 2004). It is believed that differentiation exists among Foc isolates, which may be due to long‐term interactions between Foc and cucumber.

In many areas of China, cucumbers, especially resistant cultivars, are continuously planted in greenhouses due to their high value. However, Fusarium wilt of cucumber occurs more readily in this cropping system, with an incidence ranging from 30% to 90% (Pu, Zhang, Liu, Dai, & Wang, 2011; Zhou & Wu, 2012). Many factors such as accumulation of the pathogens, deterioration of soil physicochemical properties, shift of soil microflora, and secretion of autotoxic substances are considered to contribute to the consecutive monoculture problems (Huang, Chou, & Erickson, 2006; Li, Li, Kong, Wu, & Wang, 2010; Nayyar et al., 2009; Wu et al., 2011; Yu, Shou, Qian, Zhu, & Hu, 2000). Moreover, the virulence variation of the pathogens may also play an important role. Previous research has shown that plant pathogenic fungi can adapt to the genetic background of host plant species during their interaction, and thus, form new types of virulence or physiological races (Barrett et al., 2009; Gao et al., 2012). Wang, Brubaker, Tate, Woods, and Burdon (2008) found that serial passage on a susceptible cotton cultivar for 10 generations significantly increased the virulence of *F. oxysporum* f. sp. *vasinfectum*. However, a conflicting result was found in *Verticillium dahliae* isolates, some of which showed a loss in pathogenicity after successive inoculation on the susceptible potato cultivar “Kennebec” (Alkher, EI Hadrami, Rashid, Adam, & Daayf, 2009). Gao et al. (2012) found that when *Curvularia lunata* was continuously applied to a resistant cultivar of maize under laboratory conditions, the virulence of the pathogen significantly increased. It is not clear whether successive generations of Foc on cucumber affect their virulence. Understanding how Foc responds to this continuous system is essential to minimizing fungal damage.

Generally, the variation in virulence of a pathogen is mediated by multiple genes in one or more interactive network, and various virulence‐related genes in *F. oxysporum* have been reported (Ruiz‐Roldán & Di Pietro, 2012). In the early stage of infection, genes associated with signal transduction play important roles (Jain, Akiyama, Kan, Ohguchi, & Takata, 2003; Jain, Akiyama, Takata, & Ohguchi, 2005). For example, *fga1*, which encodes a G‐protein α subunit, was confirmed to be involved in a signal transduction pathway that regulated conidiation and pathogenicity of Foc (Jain, Akiyama, Mae, Ohguchi, & Takata, 2002). Several genes encoding cell wall‐degrading enzymes (CWDEs) were found to be highly expressed in *F. oxysporum* during penetration and colonization of the host plant (Gupta, Bhar, & Das, 2013; Yadeta & Thomma, 2013). However, there was no effect on virulence when a CWDE‐encoding gene such as polygalacturonase *pg1* and *pg5*, and pectate lyase *pl1* was inactive, possibly due to functional redundancy (Di Pietro, Roncero, & Ruiz‐Roldán, 2009). Some genes associated with the regulation of CWDE genes influence the virulence of *F. oxysporum*. The functional loss of sucrose nonfermenting 1 gene *snf1* resulted in a reduction in *F. oxysporum* virulence to cabbage and *Arabidopsis* (Ospina‐Giraldo, Mullins, & Kang, 2003). Jonkers, Rodrigues, and Rep (2009) found that the inability of the *frp1*‐deficiency mutant of *F. oxysporum* f. sp. *lycopersici* (*Fol*) to penetrate and colonize plants was mainly attributed to the reduced expression of CWDE genes. When entering plants, *F. oxysporum* initiates relevant genes to breakdown host defense systems allowing it to grow on the living tissue. Deletion of the gene *chsV* that encodes a class V chitin synthase in *Fol* elicited a strong defense reaction in tomato suggesting chitin synthase may play an essential role in fungal infection (Madrid, Di Pietro, & Roncero, 2003; Pareja‐Jaime, Martin‐Urdiron, González Roncero, González‐Reyes, & Ruiz Roldán, 2010 **)**. Some virulence‐related genes of *F. oxysporum* have been shown to impact hyphal proliferation and colonization, for example, the six1 protein, an effector secreted during colonizing, is required for virulence of *Fol* (Rep, Meijer, Houterman, van der Does, & Cornelissen, 2005). However, only a few genes, including *fga1*,* fga2*, and *fgb1* encoding G‐protein α and β subunits, respectively (Jain et al., 2002, 2003, 2005) and *FocVel1* encoding a velvet protein (Li et al., 2015), have been identified as involved in Foc infection.

In this study, a Foc isolate was serially passaged on resistant and susceptible cucumber cultivars under laboratory conditions, to investigate the influence of the pressures of the host plants on fungal virulence. Four genes related to virulence of *F. oxysporum*,* fga1*,* snf1*,* frp1*, and *chsV*, were selected and their expression in Foc isolates with different virulence were assayed. This study will improve the management of cucumber Fusarium wilt and provide insight into the mechanisms underlying virulence differentiation in Foc.

## 2 MATERIALS AND METHODS

### 2.1 Strain

The original Foc strain foc‐3b used for serial passage was isolated from an infected cucumber root from an experimental field of the Institute of Plant Protection, Chinese Academy of Agricultural Sciences (CAAS) in Langfang, Hebei Province. The strain was identified based on morphological and molecular characterization (O'Donnell, Kistler, Cigelnik, & Ploetz, 1998) and determined to be a low virulent strain using pathogenicity tests (Vakalounakis & Fragkiadakis, 1999). The pure culture of foc‐3b was preserved in glycerol stocks at −80°C in the Biocontrol of Soilborne Diseases Lab of the Institute of Plant Protection, CAAS and the Agricultural Culture Collection of China (strain number: ACCC39326).

### 2.2 Cucumber cultivars

The susceptible cucumber cultivar *Cucumis sativus* L. cv. Zhongnong No. 6 (ZN6) and the moderately resistant cultivar *C*. *sativus* L. cv. Zhongnong No. 106 (ZN106) that appears slight symptoms of cucumber Fusarium wilt (Gu et al., 2008) were provided by the Institute of Vegetables and Flowers, CAAS, Beijing, China.

### 2.3 Preparation of Foc inoculum

Strain foc‐3b was incubated on potato dextrose agar (PDA, Oxoid, Hampshire, UK) in an incubator at 26°C for 5 days. Five agar blocks of mycelia were transferred into Armstrong medium containing 20.0 g of glucose, 1.6 g of KCl, 0.4 g of MgSO_4_·7H_2_O, 5.9 g of Ca(NO_3_)_2_, 1.1 g of KH_2_PO_4_, 0.2 μg of FeSO_4_, 0.2 μg of ZnSO_4_, and 0.2 μg of MnSO_4_ in 1 L distilled water (Singleton, Mihail, & Rush, 1992). The fungus was cultured at 28°C on a shaking table at a speed of 180 r/min. After 3 days, the liquid culture was passed through a 30 μm sterile mesh to remove tiny fragments of hyphae, and the microconidia were counted under a microscope (BX41, Olympus, Tokyo, Japan) using a haemocytometer. The concentration of the suspension was adjusted to 10^5^ spores/ml to prepare Foc inoculum.

### 2.4 Foc inoculation and plant cultivation

Plump cucumber seeds of resistant and susceptible cultivars were sterilized in an oven at 68°C for 3 hr, and then soaked in foc‐3b spore suspension for 10 min. The seeds were dried in the shade and sown in seed trays (30 × 15 cm, 5 × 2 holes, 5 × 5 cm/hole) filled with a mixture of vermiculite, peat and pearlite (1:1:1, v/v/v) that had been autoclaved at 121°C for 1 hr. Thirty seeds were planted for each cultivar, one seed per hole. Seeds treated with sterile distilled water were used as the control. The trays were placed in a growth chamber with a constant temperature of 26°C and a photoperiod of 16 hr light/8 hr dark (light intensity 600 μmol/m^2^ s). The treatments were arranged in a randomized complete block with three replicates.

### 2.5 Successive generations of foc‐3b on resistant and susceptible cucumber cultivars

Fourteen days after inoculation, diseased plants were identified with the symptoms of vascular discoloration and cotyledon chlorosis. The most infected cucumber seedlings were picked up to carry out the re‐isolation. They were washed with tap water thoroughly, sterilized with 3% NaClO for 3 min, and washed with sterile water 5 times. The infected roots and stems were cut into 0.2–0.5 cm pieces with a sterile scalpel, and the fragments were incubated on Pentachloronitrobenzene Peptone Agar (PPA) medium containing 15 g peptone, 1 g KH_2_PO_4_, 0.5 g MgSO_4_·7H_2_O, 750 mg pentachloronitrobenzene (PCNB) and 15 g agar in 1 L distilled water (Nash & Snyder, 1962) at 26°C for 3 days. The emerging colonies were transferred onto fresh PDA plates and incubated at 26°C for 5 days. The re‐isolated strains were identified based on their morphology and by PCR analysis of the elongation factor encoding gene *EF‐1*α (O'Donnell et al., 1998) and stored in 30% glycerol at −80°C.

Subsequently, two offspring isolates from the susceptible cultivar, Sa and Sb, and two from the resistant cultivar, Ra and Rb, were used to start the next passage using the same method described above. A total of five serial cycles were conducted for each isolate.

### 2.6 Virulence of foc‐3b offspring strains on cucumber

After five generations, the virulence of the strain foc‐3b and its offspring isolates from each cycle and each variety was assayed simultaneously in a greenhouse. Susceptible cultivar ZN6 was used as the test crop.

The inocula preparation and the seeds inoculation were carried out as described above. The inoculated seeds were dried in the shade and planted in sterile soil mixture (vermiculite: peat: pearlite = 1:1:1, v/v/v) in plastic pots (dia. 13 cm), two seeds per pot, and five pots for each isolate. The initial isolate foc‐3b was used as the control. All pots were arranged in a randomized complete block design in a greenhouse with 30/20 ± 1°C day/night and 16 hr photoperiod. The experiment was conducted three times. The disease index (DI) of Fusarium wilt was assessed 14 days after inoculation using a 5‐grade criterion (Tok & Kurt, 2010; Vakalounakis et al., 2004) with a few modifications: 0 = no symptoms; 1 = slight to moderate rot on taproots and slight wilt on cotyledons; 2 = slight vascular discoloration in stems and one cotyledon chlorisis; 3 = slight vascular discoloration in stems and two cotyledons chlorisis; 4 = severe rot on roots, vascular discoloration, and wilt of whole plant; 5 = dead seedling.

### 2.7 RNA extraction and cDNA synthesis

Expression of pathogenicity‐related genes of *F. oxysporum* was analyzed in the wild strain foc‐3b and the offspring isolates (Ra and Sa) from each generation. The strains were incubated on PDA at 26°C for 5 days. Five blocks of hyphae were transferred into Armstrong fluid and cultured at 28°C in a shaker at a speed of 180 r/min. After 72 hr, the mycelium of each isolate was collected through a sterile cheese cloth, washed 3 times with sterile distilled water to remove the medium, frozen immediately in liquid nitrogen, and kept at −80°C. The total RNA of each sample was extracted using the Trizol reagent (Invitrogen, Carlsbad, CA, USA) according the manufacturer's instruction. The concentration of total RNA was determined using an Ultraviolet Spectrophotometer (NanoDrop ND‐1000, Wilmington, DE, USA) and reverse transcribed to cDNA using a Fast Quant RT Kit (Tiangen, Beijing, China) according to the manufacturer's instructions.

### 2.8 Expression of virulence‐related genes

Four *F. oxysporum* virulence‐related genes, *fga1*,* snf1*,* frp1*, and *chsV*, associated with signal recognition, cell wall degradation, colonization, and plant defense response, respectively, were selected. Primers of the four genes were designed using the software Primer Premier 5.0 (Table 1), and their specificity was determined using PCR with the following program: 94°C for 3 min; 30 cycles of 94°C for 1 min, 60°C for 30 s and 72°C for 30 s; followed by 72°C for 10 min. The expression levels of these genes in Foc strains during vegetative growth in Armstrong medium were assayed using a SYBR Premix Ex Taq (Takara, Dalian, China) in an IQ 5 Multicolor Real‐time PCR Detection System (Bio‐Rad, Hercules, CA, USA), with synthesized cDNAs as templates and β*‐tubulin* as a reference gene (Thatcher, Gardiner, Kazan, & Manners, 2012). The reaction was performed in a 25 μl system containing 12.5 μl of SYBR Premix, 2 μl of 2× diluted cDNA, 1 μl of each primer and 8.5 μl of RNase‐free water in a 96‐well plate, conforming to the following program: 95°C for 2 min; 40 cycles of 95°C for 10 s and 60°C for 30 s. The melting curve was generated every 0.5°C from 60 to 95°C to check nonspecific amplification. The relative expression levels of the genes were calculated using the 2^–ΔΔCt^ method (Livak & Schmittgen, 2001). Three replicates were conducted for each isolate.

**Table 1:** Primers of foc‐3b used for quantitative reverse transcriptase PCR

Gene	Accession number	Primer	Position	Sequence(5′‐3′)	AT (°C)	Product size (bp)
*fga1*	AB072451.1	*fga1*‐f	557	GATCGTGGTGTGCAAGAATG	60	180
		*fga1*‐r	736	GGAGGACATCCTGGTCGTTA		
*snf1*	AF420488.1	*snf1*‐f	4,107	TTGTCCACCTCTCCTTCACC	60	177
		*snf1*‐r	4,283	GCCTGAAATGGAGCAGAAAG		
*frp1*	AY673970.1	*frp1*‐f	1,798	TCGTGGCATACTCTCGTCAC	60	180
		*frp1*‐r	1,977	CATTAGAAAGGCGAGCTTGG		
*chsV*	KC840941.1	*chsV*‐f	3,463	GGCCAAGACGTTTCCAAGTA	60	180
		*chsV*‐r	3,642	CAGGATAGATGCGAGCATGA		
β*‐tubulin*	—	*Fo*‐*Tub*‐F5	—	TGTTCGACCCCAAGAACAT	60	158
		*Fo*‐*Tub*‐R5	—	GGTCCTCGACCTCCTTCATA		

AT, annealing temperature.

### 2.9 Data analyses

The statistical software SAS 9.1.3 (SAS Institute Inc., Cary, NC, USA) was used to analyze all data. Analysis of variance (ANOVA) was used to assess pathogenicity differences between Foc offspring isolates, generations, cultivars and their interactions and the change of gene expressions using a general linear model (GLM). Mean values of three replicates were compared using Duncan's test. The relationship between virulence and gene expression level was analyzed using Pearson's correlation analysis. *p* value <.05 was considered significant.

## 3 RESULTS

### 3.1 Successive generations of foc‐3b

All 20 offspring isolates from different cultivars and generations were identified as *F. oxysporum* (data not shown) and caused Fusarium wilt on cucumber.

### 3.2 Virulence of foc‐3b offspring isolates on cucumber

**Figure 1:**
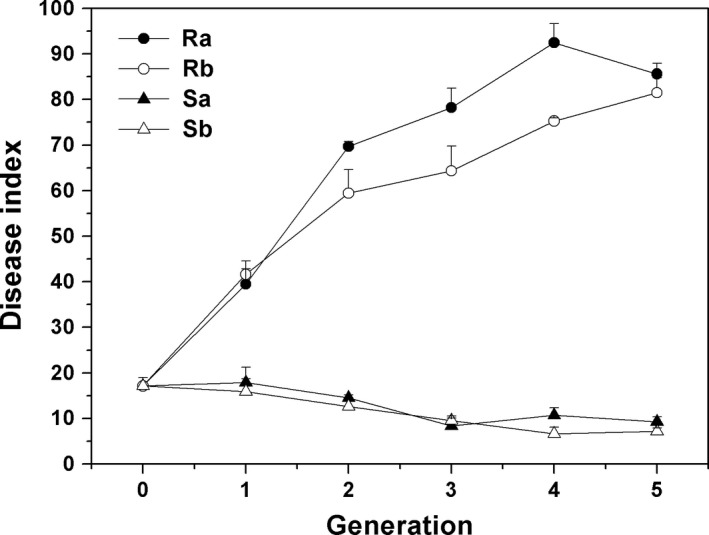
Disease index of cucumber Fusarium wilt caused by Foc strains re‐isolated from different cultivars. The seeds of susceptible cultivar ZN6 were inoculated with 10^5^ spores/ml fungal suspension and grown in sterile soil matrix in plastic pots in a greenhouse. Two seeds were planted in each pot, and five pots per replicate. All pots were arranged in a randomized complete block design with three replicates. Disease index was surveyed 14 days after inoculation using a 5‐grade criterion. Ra, Rb, and Sa, Sb represent Foc strains passed through resistant (Ra & Rb) and susceptible cultivars (Sa & Sb), respectively. Generation 0 represents the original strain foc‐3b. Data are means ± *SD* of three independent biological replicates

Most cucumber seedlings showed Fusarium wilt symptoms after inoculation with foc‐3b lineage and the disease severity varied significantly following the passage of Foc isolates through different cucumber cultivars. Cultivars showed the most significant impact on fungal disease severity, followed by cultivar × generation interaction and generation (Table 2). Disease index on susceptible cucumbers inoculated with the strains re‐isolated from the resistant cultivar was higher compared with those treated with the isolates from the susceptible cultivar and the original strain. With increasing serial passages through resistant cultivar, virulence of the strains rapidly increased, and peaked at the fourth and fifth generation. After serial passage through susceptible cucumber, disease severity significantly decreased from the third generation (*p* < .05). Though there was a difference between the two strains passed through the resistant and susceptible cultivars, respectively, their variation trends were consistent (Figure 1).

**Table 2:** Analysis of variance in disease index of cucumber Fusarium wilt caused by Foc strains serially passed through resistant and susceptible cultivars

Source of variation	Mean square	*F* value	*p* value
Cultivar	41350.39	2644.97	<.0001
Cultivar × generation	2757.51	176.38	<.0001
Generation	1557.58	99.63	<.0001
Isolate	355.88	22.76	<.0001
Cultivar × isolate	147.63	9.44	.0035
Generation × isolation	49.36	3.16	.0152
Cultivar × generation × isolate	43.96	2.81	.0263

### 3.3 Expression of the pathogenicity‐related genes and their correlation with fungal virulence

**Figure 2:**
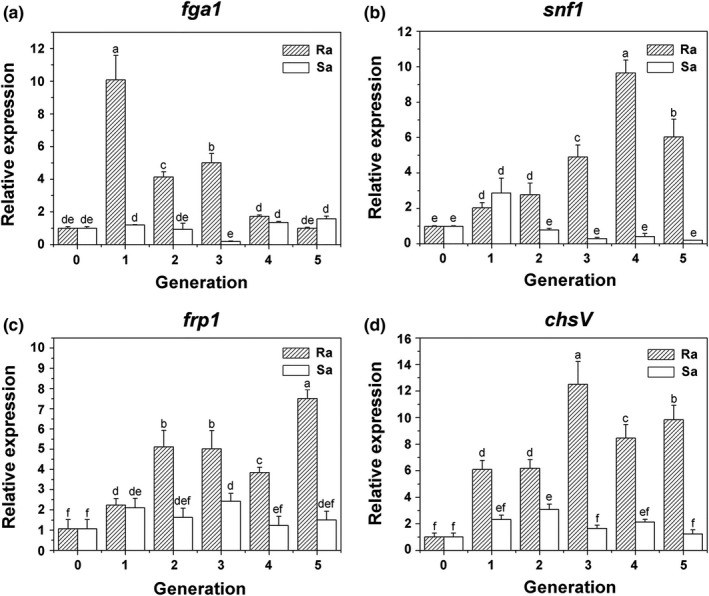
Differential expression of four genes in Foc strains serially passaged through different cucumber cultivars using quantitative reverse transcriptase PCR. (a) G‐protein α subunit encoding gene *fga1*. (b) Sucrose nonfermenting 1 gene *snf1*. (c) F‐box protein encoding gene *frp1*. (d) Class V chitin synthase gene *chsV*. Ra and Sa represent Foc strains re‐isolated from resistant and susceptible cultivars, respectively. Generation 0 represents the original strain foc‐3b. Values are means ± *SD* of three replicates

**Figure 3:**
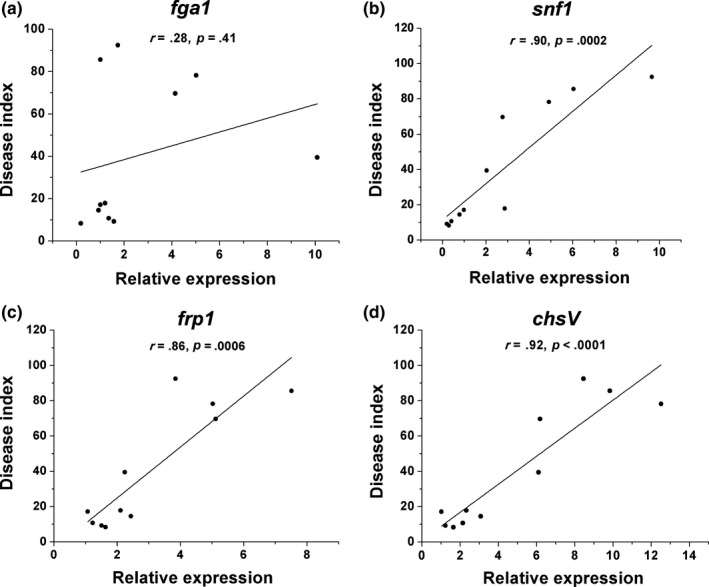
Correlation analysis between disease index of cucumber Fusarium wilt and gene expression levels in Foc strains serially passaged through resistant and susceptible cultivars, respectively. (a) G‐protein α subunit encoding gene *fga1*. (b) Sucrose nonfermenting 1 gene *snf1*. (c) F‐box protein encoding gene *frp1*. (d) Class V chitin synthase gene *chsV*. The Pearson's correlation coefficients (*r*) are calculated basing on the three replicates of foc‐3b and 10 offspring strains isolated from each generation and cultivar

The gene expression levels of *fga1* in most strains derived from the resistant cucumbers were significantly higher than those in foc‐3b and strains from susceptible plants, especially in the first generation. However, in the strains passed through the susceptible cultivar, the expression levels were consistent with the original strain, except in the third generation (*F* = 95.52, *p* < .0001, Figure 2a). No correlation was observed between *fga1* expression and Foc virulence to cucumbers (Figure 3a).

After serial passages on the resistant cultivar, the gene expressions of *snf1* in most strains were upregulated markedly compared to the original one and the strains through susceptible cucumbers. With increasing planting cycle, *snf1* expression significantly increased and peaked in the fourth generation. On the contrary, gene expression in strains from susceptible cultivar declined obviously, though it was upregulated in the first generation (*F* = 89.18, *p* < .0001, Figure 2b). A significant correlation was found between the expression levels of *snf1* and disease severity (*r* = 0.90, Figure 3b).

In strains re‐isolated from the resistant cucumbers, the relative expression of *frp1* increased with increasing serial passage, although a slight decrease was observed in the fourth generation. Expression levels increased from 2.2 to 7.5‐fold compared with the original strain. In Foc strains from the susceptible plants, *frp1* expression was upregulated at the beginning, then consistent with the original (*F* = 45.92, *p* < .0001, Figure 2c). The correlation coefficient between *frp1* expression level and disease severity was 0.86 (Figure 3c).

In the strains passed through the resistant cultivar, *chsV* expression significantly increased, especially in the third generation. In susceptible cycles, *chsV* expression levels increased in the first two generations, and then gradually declined to the level of the original strain (*F* = 77.23, *p* < .0001, Figure 2d). A significant positive correlation was found between *chsV* expression and disease severity, with a correlation coefficient 0.92 (Figure 3d).

## 4 DISCUSSION

To understand the virulence variation of Foc in continuous cropping system, we serially inoculated the strain foc‐3b on susceptible and resistant cucumber cultivars for five generations and assessed the virulence of the offspring strains.

After successive generations on different cucumber varieties, the virulence of the original strain diverged, suggesting the pathogen may adapt to specific plant hosts. When passed through the resistant cultivar, the virulence of the fungus significantly strengthened. The result was consistent with the pathogenicity of *C. lunata* which increased under the successive induction of resistant maize population (Gao et al., 2012). Under interactions with resistant cultivars, the pathogens need to overcome the biological stresses provided by the plants, thereby increased virulence is acquired during successive and long‐term planting.

The researches on susceptible plants seem to be in dispute. We found that when applied to the susceptible cucumber, the virulence of foc‐3b greatly decreased. However, for *F. oxysporum* f. sp. *vasinfectum* and *V*. *dahliae*, the virulence were increased or uncertain (Alkher et al., 2009; Wang et al., 2008). This said, in a study on virus evolution, adaptation on a susceptible cultivar was associated with a fitness cost (Montarry, Cartier, Jacquemond, Palloix, & Moury, 2012). The pathogen maintains lower virulence when continuously inoculated onto a susceptible cultivar instead of killing its host. This phenomenon may be due to the survival of the host providing more nutrition and living space for the parasite. In this study, two isolates derived from the seriously infected plants were run independently, which showed the same trends during serial cycles. More strains and field experiments are needed to verify the effects of plant hosts on the virulence of Foc.

In China, the continuous cropping system, especially of resistant cucumber cultivars, is commonly adopted in facility greenhouses, which leads to aggravated diseases. In this study, we found that the virulence of the tested strain was significantly strengthened after serial passage on the resistant cultivar and weakened on the susceptible plants, suggesting cultivar selection may play an important role in *Fusarium* virulence differentiation and rotation or mixed planting of both cultivars in continuous cucumber production may reduce or delay the incidence of Fusarium wilt. Similar cropping pattern has been successfully applied to reduce rice blast (Zhu et al., 2000). The founding may provide a new strategy for the integrated management of the fungal disease.

The virulence differentiation of a plant pathogen may be caused by the mutation of the genome structure, for example, insert, deletion, inversion, and translocation of bases. The evidence of retro‐elements and retrotransposon‐like sequences from the *gypsy* family has been reported in multiple copies of *V. dahliae* (Toshiyuki, Masahiro, Yoshiyuki, Yuta, & Yoshimiki, 2005). In *F. oxysporum* f. sp. *vasinfectum*, several mutants were identified after serial passage, based on 46 markers of amplified fragment length polymorphism (AFLP); however, no clear correlation was detected between the mutations and their virulence (Wang et al., 2008). Many studies have shown that plant pathogens co‐evolve with their hosts. However, changes to the microorganic genome may require a longer period or extreme stress. In most cases, the adaption of pathogens relies on some other processes, for example, transcriptional regulation of pathogenicity‐related genes and post‐transcriptional modification (Alkher et al., 2009; Niño‐Sánchez et al., 2015).

In this study, we demonstrated that the expressions of *snf1*,* frp1*, and *chsV*, encoding a protein kinase, F‐box protein and chitin synthase, respectively, were positively correlated with the virulence of foc‐3b offspring. Both *snf1* and *frp1* were confirmed to be involved in regulating the expression of CWDEs which can break the physical barrier of the host (Jonkers et al., 2009; Ospina‐Giraldo et al., 2003). Bidochka, Burke, and Ng (1999) and Novo, Pomar, Gayoso, and Merino (2006) also pointed out that pathogenic variability among *V. dahliae* isolates was usually associated with plant CWDEs. As a functional factor in *Fol*,* chsV* was required to overcome plant defense responses (Pareja‐Jaime et al., 2010). Here, we found the expression levels of these three genes were much higher in Foc isolates with strong virulence than those in weak isolates, suggesting that *snf1*,* frp1*, and *chsV* may play an important role in virulence variation of foc‐3b.

G‐protein α subunit‐encoding gene *fga1* has been shown to be involved in the signal transduction pathway that controls conidiation and pathogenicity of *F. oxysporum* (Jain et al., 2002). However, no relationship was found between *fga1* gene expression and foc‐3b virulence in our study. In most strains isolated from the resistant cultivar, *fga1* was upregulated. Therefore, we speculate that *fga1* might be related to virulence evolution indirectly or involved with other subunits under the selective pressure of host plants.

Besides the four pathogenicity‐related genes, *F. oxysporum* often relies on effectors (small secreted proteins) to realize colonization and successful infection (Dodds & Rathjen, 2010; Giraldo & Valent, 2013; Stergiopoulos & de Wit, 2009). Among the effectors, *six6*,* six8*,* six9*,* six11*,* six13*, and *six14* have been identified in Foc (van Dam et al., 2016); however, the expression of these genes appears to be dependent on living plant cells (van der Does et al., 2008). The expression patterns of effector‐encoding genes and other virulence‐related genes in *planta* and the function of these genes in virulence differentiation of foc‐3b require further investigation.

## 5 CONCLUSION

The empirical evidence of virulence differentiation of Foc strain foc‐3b after successive generations was presented. Virulence significantly increased after serial passage through the resistant cucumber cultivar ZN106 and weakened through susceptible cultivar ZN6. A significant positive correlation was found between the expression levels of gene *snf1*,* frp1*, and *chsV* and disease severity of cucumber Fusarium wilt, suggesting these genes may impact virulence differentiation.

## CONFLICT OF INTEREST

None declared.
